# Inhibiting the progression of arterial calcification with vitamin K in HemoDialysis patients (iPACK-HD) trial: rationale and study design for a randomized trial of vitamin K in patients with end stage kidney disease

**DOI:** 10.1186/s40697-015-0053-x

**Published:** 2015-05-01

**Authors:** Rachel M Holden, Sarah L Booth, Andrew G Day, Catherine M Clase, Deborah Zimmerman, Louise Moist, M Kyla Shea, Kristin M McCabe, Sophie A Jamal, Sheldon Tobe, Jordan Weinstein, Rao Madhumathi, Michael A Adams, Daren K Heyland

**Affiliations:** Department of Medicine, Queen’s University, 3048C Etherington Hall, Kingston, Ontario K7L 3 V6 Canada; Department of Biomedical and Molecular Science, Queen’s University, Kingston, Ontario Canada; USDA Human Nutrition Research Center on Aging, Tufts University, Boston, MA USA; Clinical Evaluation Research Unit, Kingston General Hospital, Kingston, Ontario Canada; Department of Medicine, McMaster University, Hamilton, Ontario Canada; Department of Nephrology, University of Ottawa, Ottawa, Ontario Canada; Department of Medicine, Western University, London, Ontario Canada; Women’s College Research Institute and Department of Endocrinology, University of Toronto, Toronto, Ontario Canada; Department of Medicine, University of Toronto, Toronto, Ontario Canada; Department of Medicine, Tufts University, Boston, MA USA

**Keywords:** Vitamin K, End stage kidney disease, Hemodialysis, Coronary artery calcification, Randomized controlled trial

## Abstract

**Background:**

Cardiovascular disease, which is due in part to progressive vascular calcification, is the leading cause of death among patients with end stage kidney disease (ESKD) on dialysis. A role for vitamin K in the prevention of vascular calcification is plausible based on the presence of vitamin K dependent proteins in vascular tissue, including matrix gla protein (MGP). Evidence from animal models and observational studies support a role for vitamin K in the prevention of vascular calcification. A large-scale study is needed to investigate the effect of vitamin K supplementation on the progression of vascular calcification in patients with ESKD, a group at risk for sub-clinical vitamin K deficiency.

**Methods/Design:**

We plan a prospective, randomized, double-blind, multicenter controlled trial of incident ESKD patients on hemodialysis in centers within North America. Eligible subjects with a baseline coronary artery calcium score of greater than or equal to 30 Agatston Units, will be randomly assigned to either the treatment group (10 mg of phylloquinone three times per week) or to the control group (placebo administration three times per week). The primary endpoint is the progression of coronary artery calcification defined as a greater than 15% increase in CAC score over baseline after 12 months.

**Discussion:**

Vitamin K supplementation is a simple, safe and cost-effective nutritional strategy that can easily be integrated into patient care. If vitamin K reduces the progression of coronary artery calcification it may lead to decreased morbidity and mortality in men and women with ESKD.

**Trial registration:**

NCT 01528800.

## Abrégé

**Contexte:**

La maladie cardiovasculaire, qui est partiellement attribuable à la calcification vasculaire progressive, est la cause principale de décès chez les patients atteints d’insuffisance rénale terminale (IRT) en hémodialyse. La vitamine K pourrait jouer un rôle dans la prévention de la calcification vasculaire, en raison de la présence de protéines dépendantes à la vitamine K dans les tissus vasculaires, dont l’ostéocalcine. Le modèle animal, de même que des études d’observation, témoignent du rôle de la vitamine K dans la prévention de la calcification vasculaire. Une étude réalisée à grande échelle serait nécessaire afin d’étudier l’effet de la supplémentation de vitamine K sur la progression de la calcification vasculaire chez les patients atteints d’IRT, un groupe à risque pour les carences infracliniques en vitamine K.

**Méthode/type d’étude:**

Nous prévoyons effectuer un essai clinique aléatoire, à double insu et multicentrique auprès de patients atteints d’IRT, traités en hémodialyse hospitalière en Amérique du Nord. On affectera au hasard les sujets admissibles qui présentent dans l’artère coronaire un taux de calcium supérieur ou égal à 30 unités d’Agatston, soit au groupe auquel on administre un traitement (10 mg de phylloquinone trois fois par semaine), soit au groupe témoin (administration du placebo trois fois par semaine). Le critère d’évaluation principal est la progression de la calcification de l’artère coronaire, définie comme une augmentation supérieure à 15% (résultat CAC) par rapport au point de référence, au bout de douze mois.

**Discussion:**

La supplémentation de vitamine K est une stratégie nutritionnelle à la fois simple, sécuritaire et rentable, qui peut être aisément intégrée aux soins aux patients. S’il s’avère que la vitamine K réduit la progression de la calcification de l’artère coronaire, il pourrait y avoir une diminution de la morbidité et de la mortalité chez les hommes et les femmes atteints d’IRT.

## Background

At least 2 million Canadians have chronic kidney disease (CKD) and over 20,000 Canadians require life-sustaining hemodialysis (HD) treatment for end stage kidney disease (ESKD) [1]. This important health problem carries a high burden of cardiovascular disease-related mortality. The elevated risk of cardiovascular disease in men and women with ESKD is due, in part, to the consequences of vascular calcification [2]. The Kidney Disease: Improving Global Outcomes (KDIGO) Clinical Practice Guidelines for the management of the CKD-mineral bone disorder recommend screening patients for the presence of vascular calcification but had no specific recommendations for the treatment or prevention of vascular calcification [3].

In patients with ESKD, vascular calcification is associated with abnormalities in divalent ion metabolism (e.g. phosphate homeostasis) and accompanied by alterations in bone turnover and increased fracture risk. Vitamin K is a class of fat-soluble nutrients that function as a co-factor for the enzyme γ-glutamyl carboxylase (GGCX), which activates a number of vitamin K-dependent proteins that are involved in the inhibition of vascular calcification (e.g. matrix Gla protein (MGP)) and maintenance of bone health (e.g. osteocalcin). MGP is a local inhibitor of vascular calcification that is present in the artery media. MGP requires the vitamin K dependent γ-carboxylation of its Gla residues in order to acquire calcium binding activity [4]. Previous studies demonstrate a critical role of vitamin K in modifying vascular calcification in animals with CKD [5-9]. Further, patients across the spectrum of CKD have a high prevalence of vitamin K deficiency and studies consistently indicate that patients with ESKD are those at greatest risk [7,8,10]. Therefore, vitamin K may play a key role in the development and progression of the calcification abnormalities that are frequently observed in patients with ESKD.

KDIGO research recommendations state that ‘studies are needed that compare patient outcomes of specified treatment strategies in response to the presence or absence of vascular calcification’ [3] and specifically recommends ‘to determine the efficacy of calcification inhibitors in the prevention or delay of arterial calcification, a prospective, randomized, placebo-controlled trial evaluating the administration of vitamin K in CKD stages 4-5D (dialysis) should be conducted’.

As a result of these recommendations and the potential role of vitamin K in vascular calcification in ESKD we designed the Inhibit the Progression of Arterial Calcification with vitamin K in Hemodialysis Patients (iPACK-HD), a randomized trial designed to determine whether supplementation with phylloquinone (vitamin K1) prevents the progression of coronary artery calcification in patients starting hemodialysis. We hypothesize that vitamin K supplementation will inhibit the progression of coronary artery calcification, measured by coronary artery calcification (CAC) scores, in patients with ESKD treated with HD.

## Methods/Design

### Overall study aims

The primary aim of iPACK-HD is to determine if vitamin K in the form of phylloquinone (10 mg three times per week) decreases the progression of coronary artery calcification, as measured by high resolution computed tomography over 12 months, compared to placebo. The secondary aim is to determine if phylloquinone (10 mg three times per week) decreases the incidence of vertebral fracture over 12 months, compared to placebo.

### Design

The iPACK-HD trial is a randomized, double-blind (subjects and physicians), placebo-controlled, Phase II, multicenter study which will be conducted in North America (Canada and United States). In this study, incident (within 6 months) hemodialysis patients with ESKD are randomized to receive 10 mg of phylloquinone three times per week or matching placebo after each dialysis session for 12 months.

### Ethical considerations

Our study has been (protocol and informed consent) approved by the Queen’s University and Affiliated Teaching Hospitals Research Ethics Board (Kingston, Canada). The study has been approved by Health Canada. Written informed consent will be obtained each subject. The trial is registered at clinicaltrials.gov (NCT01528800).

### Inclusion criteria

Participants in the trial must be adult patients (≥18 years of age) who have ESKD on the basis of irreversible chronic kidney disease and are new to hemodialysis treatment (<6 months). Subjects meeting inclusion criteria will be approached by a trained research associate and, after obtaining informed consent, undergo a high resolution CT scan to obtain a coronary artery calcium (CAC) score. Subjects will be eligible for randomization if they have a CAC score of ≥ 30 Agatston Units (AUs). Justification for including only those with a CAC score of ≥ 30 AUs include: 1) 70% of incident HD patients already have calcification; 2) studies have demonstrated that HD patients with a CAC score < 30 AU are unlikely to progress over 12 months [11,12]; 3) patients with high CAC scores are expected to have a faster rate of progression allowing us to detect a difference with the intervention over a shorter period of time [13] and 4) measurement of CAC scores ≥ 30 AUs have been shown to have high reproducibility in dialysis patients [14].

### Exclusion criteria

We will exclude subjects who: 1) are receiving warfarin; 2) have previous coronary artery bypass grafting or have coronary stents because of difficulty with CAC score interpretation; 4) are pregnant, breast feeding, or unwilling to practice abstinence or contraception; 5) have other severe co-morbid conditions (e.g. malignancy, disabling stroke) and are not expected to survive one year; 6) are currently enrolled in another interventional trial in which the treatments or outcomes in that trial would influence the results of the current study; 7) are unable to provide signed informed consent.

### Random allocation of patients

After obtaining informed consent, the participant will undergo a CT scan to determine CAC score. If the CAC score is ≥ 30 AUs, randomization is performed using a web-based central randomization system at the Clinical Evaluation Research Unit at Kingston General Hospital. Randomization is concealed and is stratified by centre and diabetes mellitus status using permuted blocks of random size. The prevalence of CAC is significantly higher in patients with diabetes mellitus therefore it is critical that the groups be balanced for this risk factor [15]. A designated research pharmacist(s) will access the secure web-based centralized randomization system and obtain the patient’s treatment allocation. The research pharmacist will prepare the study medication according to the assigned treatment allocation. The study drug will be will be dispensed to the HD unit and will be administrated post-dialysis to the subject. The research pharmacist will be the only individual who is aware of the treatment assignment.

### Intervention

Patients will receive either 10 mg of phylloquinone or matching placebo that is identical in sight, taste and smell, following each dialysis treatment three times/week for a total of 12 months. Phylloquinone is the only available form of vitamin K for clinical use in Canada and the United States*.* It is currently used for the prevention of hemorrhagic disease of the newborn, for the reversal of anticoagulation, and in patients with impaired absorption of fat-soluble vitamins (e.g. cystic fibrosis). Although certain forms of vitamin K2 (e.g. menaquinone-4) are available as health food supplements and/or used clinically in some countries, phylloquinone is the form most commonly consumed in the diet and used clinically in North America with established efficacy and safety. The single RCT performed in humans that similarly considered a CAC score as an endpoint studied phylloquinone [13]. Furthermore, phylloquinone decreased calcification in rats with experimental CKD. The dose of 10 mg thrice weekly dose (30 mg/week) was chosen based on the experience of the Vitamin K Supplementation in Post-menopausal Women with Osteopenia (ECKO trial) where a similar weekly dose was administered over 4 years in Canadian post-menopausal women [16] and also based on pre-clinical studies demonstrating that a 100-fold increase in phylloquinone content in rodent chow decreased the incidence and severity of vascular calcification [9]. Problems with polypharmacy and adherence to medications are both well documented in dialysis patients therefore administration of the 10 mg dose of phylloquinone will be after each HD treatment to improve adherence. A placebo control group is justified because phylloquinone supplementation is not part of standard clinical care, has not been shown to improve important outcomes and has not been routinely recommended in the management of HD patients.

### Blinding and protection against bias

At the time of randomization into the study, treatment assignment is concealed from the local research team, hemodialysis care team, subjects and families. Subjects are randomized to receive either phylloquinone or a matching placebo that is identical in appearance, and they, their families, their clinical and research teams will remain blinded to allocation. Recording of baseline data and relevant clinical follow-up data will be performed by study personnel who are blinded to treatment allocation (Table 1).

**Table 1:** **Baseline and ongoing data collection**

Baseline data	age, sex, ethnicity, height, target weight, waist circumference
	dialysis start date, cause of ESKD
	type of dialysis access
	medication use
	comorbidities
	hemoglobin, albumin, Kt/V, urea reduction ratio, lipid profile
	calcium, phosphate, PTH, alkaline phosphatase
	vitamin K status (phylloquinone, PIVKA-II, osteocalcin)
Monthly data	Hemoglobin, albumin, Kt/V, urea reduction ratio
	Calcium, phosphate
	Hospitalizations
	Cardiovascular events and procedures
	Medication use
	Adherence
Q3 monthly laboratory data	PTH, alkaline phosphatase, lipid profile
Q4 monthly laboratory data	Vitamin K status (phylloquinone, PIVKA-II, osteocalcin)

### Outcomes

#### Primary endpoint

The primary objective of the iPACK-HD trial is to investigate the effect of vitamin K on the progression of coronary artery calcification in patients with ESKD. The primary outcome will be the progression of coronary artery calcium (CAC) score at 12 months as defined by an increase of 15% or more from the CAC score measured at baseline. The coronary calcium scoring protocol is scanned during a single breath hold with contiguous slice acquisition at a thickness of 2.5 mm. Imaging is acquired from the apex of the heart to the inferior margin of the aortic arch. CAC will be measured using the standard Agatston/Janowitz protocol in the left main, left anterior descending artery, circumflex artery and right coronary artery and composite and the overall CAC score is calculated using a weighted value assigned to the highest density of calcification in a given coronary artery. The scans will be scored by one individual blinded to the subject and treatment allocation. From the time of randomization, treatment duration will be 12 months. Based on the report of Block *et al.* there is a significant increase in CAC score at 12 months in patients new to HD [11]. The median increase in CAC score at 12 months in patients randomized to a calcium-based phosphate binder was 142 AU (median % increase of 41%). Two landmark RCTs conducted in dialysis patients that evaluated two different regimens (e.g. sevelemer and cinecalcet compared to calcium and placebo respectively) demonstrated significant treatment effects on the progression of CAC at 12 months [17,18]. As such, the 12 month time frame should be sufficient to demonstrate a treatment effect of vitamin K should one exist.

A CAC score change (either increase or decrease) greater than 15% is considered evidence of true change as opposed to scoring variability [19]. In the Multi-Ethnic Study of Atherosclerosis, a graded relationship of CAC progression with coronary heart disease event risk was demonstrated, with greater CAC progression associated with greater risk [20]. The authors examined the threshold at which annualized relative (percent change) progression was best associated with coronary heart disease events and a greater than 15% annual increase (HR: 1.4, p < 0.05) in CAC score was identified. An RCT of an intervention that differs from iPACK-HD - the use of sevelamer rather than calcium as a phosphate binder - showed a 30% risk reduction for CAC progression (defined as an increase of >15% in CAC score) [11]; participants in this trial were later shown to have experienced a 60% reduction in the risk of death [21]. These data support the clinical relevance of this threshold.

The definitive trial to determine the impact of vitamin K on cardiovascular disease in this population would examine calcification-sensitive cardiovascular outcomes. However, before moving directly to a large scale, very expensive, Phase III trial powered to examine the treatment effect on cardiovascular events, we will first conduct first a Phase II study using CAC scores as a surrogate outcome. Evidence for an adequate surrogate is often considered under the framework of the Prentice criteria. For a given treatment: 1) the treatment affects the outcome; 2) the treatment affects the surrogate; 3) the surrogate affects the outcome; 4) the effect of the surrogate on the outcome is independent of the treatment. For the surrogate outcome of vascular calcification, a subgroup analysis of an RCT of phylloquinone- the intervention that we propose to study - resulted in significantly less progression of CAC over 3 years in low-risk elderly people whose baseline CAC score was ≥ 10 AUs [13]. An RCT of an intervention that differs from this study - the use of sevalamer rather than calcium as a phosphate binder - showed a 30% risk reduction for CAC progression (increase of >15% in CAC score over 18 months) [11]; participants in this trial were later shown to a 60% reduction in the risk of death [21]. In an observational study, CAC progression strongly predicted myocardial infarction (criterion 3) [22]. If CAC score is not affected by the intervention, we will regard it as unlikely that the intervention affects patient-important cardiovascular outcomes since our information about its effects suggest mechanisms that operate through vascular calcification.

#### Secondary end pointes

We will evaluate 3 secondary endpoints at 12 months: (1) the incidence of vertebral fracture will be based on patient report with adjudicated review of source documentation and anterior and lateral radiographs of the thoracic and lumbar spine at baseline and at 12 months. Vertebral fractures will be defined as prevalent and/or incident using a standard methodology, vertebral morphometry, in accordance with the recommendations of the National Osteoporosis Foundation Working Group on Vertebral Fracture; (2) clinical outcomes including mortality, cardiovascular events (acute coronary syndrome, congestive heart failure, stroke, transient ischemic attack, amputation; cardiac, cerebral or peripheral revascularization procedure (bypass, angioplasty, amputation, stents), thrombosis (deep vein thrombosis, pulmonary embolism, dialysis access thrombosis (fistula, graft or catheter), causes and lengths of hospitalization and bone fracture-related events and (3) changes in biomarkers of vitamin K status: phylloquinone, and the carboxylation status of the vitamin-K-dependent proteins (VKDPs) osteocalcin, and prothrombin.

### Data collection and follow-up procedures

Patients will be followed from study randomization until study completion (12 months study period) or patient censor (see below for details). The study coordinator will review each patient and their records each month. A web-based electronic case report form for data entry will be used. Baseline and ongoing data collection plans are presented in Table 1.

Co-interventions will be documented each month. It is not practical to standardize all these treatments and doing so would limit the generalizability of this study. All study participants will receive a conventional HD prescription (~4 hours thrice weekly, high flux dialysis membrane). Hypertension and dyslipidemia will be managed according to the participants’ treating physicians. Patients may receive a general or dialysis-specific (B and C vitamins) multivitamin preparation. The chronic kidney disease associated abnormalities in mineral metabolism (e.g., hyperphosphatemia, hyperparathyroidism) will be managed by attending nephrologists according to local clinical practice informed by guidelines [23]. Usual practice targets and the currently accepted Canadian Society of Nephrology quality assurance indicators for HD patients are as follows: calcium (2.2-2.5 mmol/), phosphorus (<1.8 mmol/L) and PTH (10–50 pmol/L). The majority of HD patients in Canadian centers receive elemental calcium as their primary phosphate binder. The American Kidney Disease Quality Outcomes Initiative (K/DOQI) clinical practice guidelines recommend targeting a similar phosphate level (<1.78 mmol/L) but suggest limiting the total daily dose of elemental calcium to 1500 mg/day (evidence level: opinion) [24]. Calcitriol (an active form of vitamin D) is frequently prescribed, and cinecalcet (a calcimimetic) is prescribed to a limited extent, for the management of secondary hyperparathyroidism. Based on current understanding, phylloquinone treatment would not be expected to alter levels of calcium or phosphate and therefore the use of important co-interventions should not be changed by treatment allocation.

### Study censoring

As the primary endpoint requires completion of the CT scan, follow up for this outcome can only occur if the patient has survived 12 months. Patients will be censored and withdrawn from follow-up at the following points in time: development of an indication for use of warfarin treatment, kidney transplantation, transfer of patient to another city which is not a satellite of the original study centre, and death. With the exception of death, we will obtain CAC scores at the time of censoring (within a window of one month before or after the date of censoring), and use this as a last observation carried forward, in one of our missing data sensitivity analyses. Loss to clinical follow up is very unlikely in this group of patients who are dependent on life-sustaining therapy. When patients undergo coronary stenting, the CAC score can no longer be used to assess calcification in the stented arteries. In these subjects, we will compare calcification scores from available arteries at follow up with scores from the same arteries at baseline.

### Statistical analysis

#### Justification of sample size

The primary endpoint is the progression of coronary artery calcium (CAC) score at 12 months as defined by an increase of 15% or more from baseline. Sample size calculations are based on the study of Block *et al.* In this study, 109 incident HD patients were randomized to one of two phosphate binders (calcium versus sevelamer) and CAC progression (defined as a 15% increase in CAC score) in patients with a baseline CAC score of ≥ 30 AUs was determined at 12 months [11]. The proportion of patients reaching the primary outcome at 12 months was 86% in patients randomized to calcium and 44% in patients randomized to sevelamer. The majority of hemodialysis patients receive calcium as the sole phosphate binder (82% in Canada) or receive 1500 mg of calcium daily in combination with another binder. Therefore, for the purpose of sample size estimation we conservatively expect the progression rate in our placebo arm will be lower than the calcium arm of the Block study (86%). Further, we conservatively estimate that the addition of phylloquinone will have a more modest reduction in progression than the calcium-sparing sevelemer arm of the Block study (44%). Therefore, to achieve 80% power using a two-sided (alpha = 0.05) Chi-Squared test to detect a decrease in CAC progression over 12 months from 70% to 56%, or a relative risk reduction of 20%, we need to enrol 186 patients per arm (Power and Sample Size 8, Kaysville, Utah) (see Table 2). Therefore, we will enroll a total of 448 patients which conservatively accounts for up to 20% loss to trial completion by 12 months.

**Table 2:** **Sample size justification**

**Control arm CAC progression rate**	**Relative risk reduction**	**Phylloquinone arm progression rate**	**Sample size per arm for 80% power**
80	20%	64%	123
**70**	**20%**	**56%**	**186**
60	20%	48%	270
50	20%	40%	388
80	25%	60%	80
70	25%	53%	121
60	25%	45%	173
50	25%	38%	247
RRR = Relative risk reduction			

Estimate of the power for testing the effect of the intervention. The effect sizes are described in terms of relative risk reduction for a > 15% increase in CAC score over baseline in 12 months. The bolded row indicates estimates utilized for sample size calculation for the iPACK-HD trial.

### Statistical analysis

Analysis of the primary endpoint will be based on the intention-to-treat principal. The primary analysis is the between-arm comparison of the proportion of patients whose CAC score increases by 15% or more from baseline to 12 months using relative risks and 95% confidence intervals. This comparison will employ the Mantel-Haenszel Chi-Squared test stratified by centre and diabetes mellitus (the pre-stratification factors). The primary analysis will not adjust for additional covariates, but a sensitivity analysis will employ logistic regression stratified for centre and diabetes mellitus and including any covariates that demonstrate clinically important imbalance at baseline. A secondary analysis will examine the change in continuous CAC score adjusting for baseline CAC score and covariates. In a sensitivity analysis, the composite outcome of death or progression of CAC will be examined. In the unexpected event that more than 20% of patients are lost to follow-up or die we will employ multiple imputations on the continuous CAC scores prior to dichotomizing it for analysis. With respect to the secondary endpoints, we will report the fracture rate, and the absolute difference in rates, by arm with exact binomial 95% confidence intervals. Due to the limited sample size, we will similarly not test for differences in clinical events however these will be reported by arm with exact bimodal 95% confidence intervals.

### Pilot study

Before advancing to the large-scale trial, a randomized, double-blind, placebo controlled pilot study of 60 patients is being carried out to determine the feasibility of a multicenter RCT. The primary focus of the pilot study is to evaluate recruitment of trial patients, adherence to protocol and completeness of outcome assessment data. After completion of the pilot trial, we will initiate the definitive randomized, double-blind, placebo controlled multicenter trial to evaluate the overall study hypothesis.

## Discussion

The presence and extent of vascular calcification as well as its progression is a significant predictor of cardiovascular morbidity and mortality in patients with ESKD. Thus, therapeutic strategies that alter the development or progression of vascular calcification are likely to be of benefit. To date, randomized controlled trials (RCTs) have focused on treatments that reduce blood levels of phosphate (e.g. phosphate binders). The change in CAC score over time has been used as the primary endpoint in all of these studies [11,17,25-27] and is the primary endpoint for this proposed trial.

Vitamin K, based on its ability to enhance the function of MGP as a key vascular calcification inhibitor, is one potential strategy to reduce the progression of vascular calcification in patients with ESKD. Vitamin K exists naturally in two molecular forms - vitamin K1 (phylloquinone) and vitamin K2 (menaquinones) [28]. Phylloquinone is the major dietary form, whereas menaquinones, with the exception of menaquinone-4, are believed to be produced by gut bacteria. The extent to which these manquinones contribute to overall vitamin K status is unknown. However all forms of vitamin K catalyze the carboxylation of VKDPs [28,29]. The trials that have evaluated the impact of vitamin K supplementation on calcification outcomes have utilized phylloquinone. Similar results of menaquinone interventions have not been reported.

MGP is synthesized by VSMCs and requires vitamin K dependent γ-carboxylation of its specific Glu domains to actively inhibit calcification [4]. Results of *in vitro* experiments suggest MGP becomes up-regulated adjacent to sites of calcification to presumably limit the progression of vascular calcification in a negative feedback manner [30]. Furthermore, marked MGP up-regulation has been demonstrated *in vivo* in aortas of animals with experimental CKD [31] yet the higher tissue concentrations of MGP resulted predominantly from accumulation of the uncarboxylated form (ucMGP). These data indicate that the regulation of MGP expression, as well as its activity, becomes altered in the CKD environment.

Results of preclinical studies demonstrate that a therapeutic dose of warfarin depletes tissue vitamin K concentrations and markedly increases the susceptibility of all vessels to vascular calcification in an adenine rat model of CKD [9]. Furthermore, high dietary phylloquinone significantly increases tissue phylloquinone concentrations and reduces the incidence and severity of vascular calcification in rats with CKD. These data indicate that, in the setting of experimental CKD, vitamin K status is important to the inhibition of vascular calcification. Therefore, factors that increase vitamin K availability to the vasculature may be critical to MGP γ-carboxylation and the inhibition of vascular calcification in patients with CKD.

To date, no trial has examined whether vitamin K supplementation prevents the progression of coronary artery calcification in patients with chronic kidney disease, a group in which high risk has been established. Studies have consistently demonstrated that vitamin K deficiency is prevalent in HD and peritoneal dialysis patients, as well as in individuals with earlier stages of CKD [8-10,32,33]. Furthermore, 82.5% HD patients have low vitamin K status and this has been attributed to a lower dietary intake compared to healthy individuals [34]. While the exact reason for the low intake is unknown, it may be related to the dietary regimen prescribed for HD patients. Preliminary studies in humans have demonstrated that ucMGP levels augment progressively with CKD stage and are independently associated with aortic calcification and mortality [35]. Taken together, there is considerable evidence to suggest that patients with kidney failure are at risk for pathophysiological consequences of suboptimal vitamin K status.

The studies that have evaluated the impact of vitamin K supplementation on calcification in patients not at high risk for vitamin K deficiency and vascular disease have been positive. In an RCT of healthy adults 60–80 yrs old, supplementation with daily phylloquinone (0.5 mg/day) slowed the progression of CAC over 3 years among those with pre-existing CAC at baseline (AUs > 10) [13]. Similarly, in an earlier RCT, post-menopausal women who were randomized to receive a supplement containing 1 mg/day phylloquinone in addition to minerals and 320 IU vitamin D3 had better carotid artery distensibility, compliance, and elasticity after three years, compared to women who received the mineral supplement alone or the mineral supplement with vitamin D3 [36].

Clinical trials of vitamin K supplementation to date have studied individuals who were not at any particular risk for sub-clinical vitamin K deficiency and had no history of clinical cardiovascular disease. No trials have been performed in an at-risk patient group such as those with kidney disease. There are no national guidelines that address an optimal intake of vitamin K outside of HD patients receiving prolonged antibiotic therapy. A simple nutrient strategy that modifies arterial calcification could reduce morbidity and save lives.
